# Influence of Trajectory and Dynamics of Vehicle Motion on Signal Patterns in the WIM System

**DOI:** 10.3390/s21237895

**Published:** 2021-11-26

**Authors:** Artur Ryguła, Andrzej Maczyński, Krzysztof Brzozowski, Marcin Grygierek, Aleksander Konior

**Affiliations:** 1 Department of Transport, Faculty of Management and Transport, University of Bielsko-Biala, Willowa 2, 43-300 Bielsko-Biała, Poland; amaczynski@ath.eu (A.M.); kbrzozowski@ath.eu (K.B.); 2 Department of Geotechnics and Roads, Faculty of Civil Engineering, Silesian University of Technology, Akademicka 5, 44-100 Gliwice, Poland; marcin.grygierek@polsl.pl; 3 APM PRO, Barska 70, 43-300 Bielsko-Biała, Poland; aleksander.konior@apm.pl

**Keywords:** weigh-in-motion, piezoelectric sensors, strain-gauge sensors, inductive loops

## Abstract

This paper presents the analyses of the signals recorded by the main sensors of a WIM test station in the cases of abnormal runs (i.e., runs with the changes of trajectory or the dynamics of vehicle motion). The research involved strain gauges which are used for measuring the weight of vehicles, inductive loops, as well as piezoelectric sensors used, inter alia, to detect twin wheels and to determine where a vehicle passes through a station. Since the designers intend the station to be able to implement the direct enforcement function, the selection of runs deviating from the normative ones constitutes an important issue for the assessment of the measurement reliability. The study considered the location of the trajectory of the runs, the dynamics (acceleration/braking) and the trajectory changes. The change in the amplitude and the value of the signal recorded by the strain gauges as a function of the location (position) of the contact between sensor and tires is a noteworthy observation which indicates the need to monitor this parameter in automatic WIM systems. Other tests also demonstrated the influence of the analysed driving parameters on the recorded results. However, by equipping the WIM station with a set of duplicate strain gauges, the measurement errors of the gross weight and axle loads are normally within the accuracy limits of class A(5) stations. Only in the case of accelerating/decelerating, does the error in measuring the load of a single axle reach several per cent.

## 1. Introduction

Weigh-In-Motion (WIM) systems are in many countries the primary source of information about overloaded vehicles on the road. They allow to select such vehicles quickly while providing a range of other relevant traffic data. The data recorded by the system of sensors and measuring devices included in the WIM station are also a source of data on key traffic parameters such as the volume, density, and traffic speed. This information enables complex analyses and the construction of predictive models [1,2]. WIM systems can also find application in assessing the environmental impact of road transport [3], or the occupancy level of public transport vehicles [4]. The increasing requirements placed on them have resulted in a great deal of research and development work being carried out all over the world both in terms of the concept of WIM systems themselves and their individual components. A relatively new issue is the development of WIM systems adapted for direct enforcement (i.e., systems directly delivering penalties) [5].

High-speed WIM systems use sensors based on different technologies. The design and performance characteristics of the most used are reported, among others, in the article [6]. These include piezo-polymer, piezo-quartz, bending plate, and single load cell sensors. In addition, sensor readings are affected by various environmental factors. In [7], the dependence of weighing results on pavement temperature and vehicle speed for polymer, quartz, and plate sensors is presented. In [8] a recommendation was formulated that in the case of using polymer sensors, the system should be equipped with algorithms to compensate the influence of temperature changes on the weighing result, e.g., temperature correction or auto-calibration (according to the author, quartz sensors are practically not susceptible to changes in surface temperature). However, it should be noted that in recent years research related to fibre-optic sensors has also been ongoing. An up-to-date overview of sensors made with this technology can be found in [9], with a discussion concerning mainly pavement monitoring systems. The paper [10] presents the results of the initial part of a project aimed at developing a new system for measuring mass in motion based on the use of an optical sensor. The authors presented various optical measurement methods. Another concept currently under development is a solution using multiple (several dozen) point sensors (disks). Such a solution consisting of 56 sensors evenly distributed in four rows is presented in [11].

A number of works point out that not only the type of sensor used, but also the quality of installation and, above all, the condition of the road surface are crucial for measurement accuracy. In addition, several ways to reduce errors recorded by WIM systems have been proposed in the field literature. One of them is the use of an appropriate sensor signal processing algorithm. In [12], selected algorithms for processing piezoelectric sensor signals are discussed. The peak voltage, the area under the signal graph and re-sampling algorithms were mentioned. At the same time, it was pointed out that these algorithms do not solve, for example, the problem of driving against and with the wind. Yet another direction aimed specifically at reducing the impact of dynamically varying wheel loads is the multi-sensor WIM installation (MS-WIM) which provides multiple measurements of the instantaneous load on each axle. Research is being conducted on the application of various algorithms in MS-WIM systems to reduce errors due to motion dynamics and the spatial repeatability of axel dynamics [13,14,15,16,17]. 

By contrast, the article [18] points out that neural networks can identify underlying relationships, such as the spatial repeatability in axle dynamics, and that they can efficiently remove noise. Furthermore, unlike conventional weigh-in-motion calibration algorithms, they can adapt to changing circumstances, such as traffic characteristics, road profile, or sensor failure. A neural network was used to improve the measurement accuracy in the aforementioned multi-sensor system [11]. However, the need to collect extensive learning and testing sets, which can be very difficult in real WIM systems, is a significant drawback of neural networks. This is because it would require each vehicle learning and testing the network to be weighed under static conditions [13]. 

The literature does not particularly deal with the detection of where the wheel passes the sensor, the determination of the tyre footprint width or the detection of twin tyres. These issues are almost exclusively addressed by authors working on fibre-optic sensors. The publications [19] and [20] focus on the problem of estimating tyre width using a fibre-optic sensor (FOS) measurement system. 

To complement the state of the art, this study undertook the task of analysing the signals recorded by the individual sensors of the WIM system in the case of abnormal runs (i.e., significantly different from typical). The novelty of this paper is the description of the analysis of the results obtained from an experiment involving the changes in trajectory and the dynamics of a vehicle which was performed in real traffic conditions on a test station. It is also the first step of building new functionality of assessing the reliability of the WIM measurement. The experiment was planned and carried out using two categories of heavy-duty vehicles to evaluate changes in the characteristics of measurement signals generated under such conditions. The research was carried out on a WIM test station meeting all the quality criteria defined in COST 323 [21]. A series of rectilinear runs asymmetrical with respect to the lane axis, runs with a significant change in dynamics (acceleration/braking) as well as runs with a significant change in trajectory was conducted as part of the study. The signals generated by strain gauges, piezoelectric sensors, and inductive loops were analysed. The waveforms of the recorded signals and selected measures characterising the obtained signals are presented. Reference is also made to quantities, such as total weight, vehicle length, and axle loads. The influence of the conditions of the tested runs on the waveforms of the recorded measurement signals was assessed, in particular, by comparing the values of the measures characterising the signal with the measures obtained for the waveforms of these signals recorded for runs considered by the authors as normative.

## 2. Materials and Methods

### 2.1. Location

**Figure 1:**
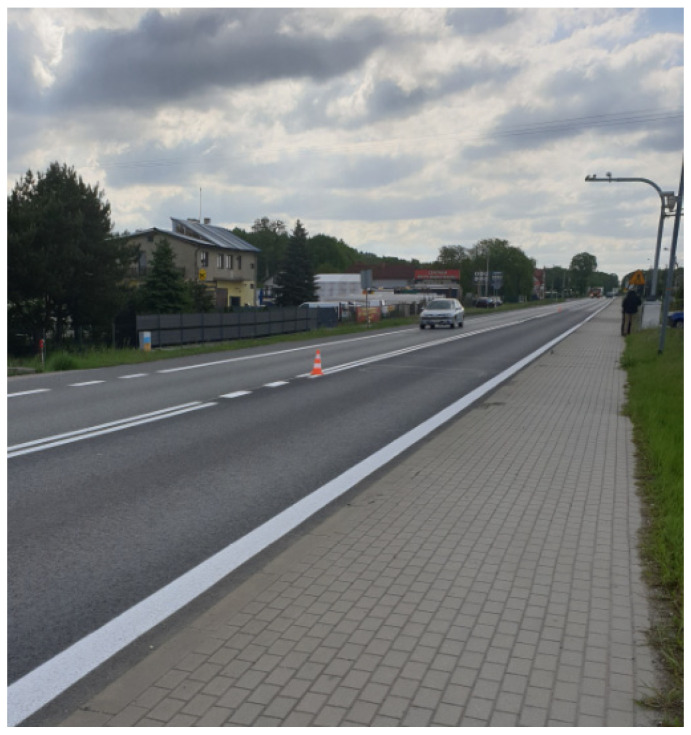
WIM station located on the DK44 road.

The WIM test station is located in Poland on the DK44 single carriageway national road in the Silesia region (Figure 1). Information on annual average daily traffic (ADDT), the proportion of heavy goods vehicles (HVs) and lane widths is shown in Table 1.

Prior to the commencement of the tests, the technical condition of the pavement was assessed in terms of the requirements for the operation of the WIM station. This assessment was based on the recommendations of COST 323 [21] regarding the suitability of the section for the operation of a pre-selective vehicle weighing system in pavement quality site class I (WIM site I Excellent) for class A(5) and B+(7) measurement systems (Table 2). The assessment involved pavement deflection measurements, longitudinal evenness measurements, and rut depth measurements.

The DK44 road in the section in question, i.e., 50 m before and 25 m after the location of the WIM station, is a straight section. The values of the longitudinal and transverse gradients of the road and the evenness of the pavement are shown in Table 3. The presented measurement results confirm that the section in question meets the requirements of COST 323 for site I Excellent.

**Figure 2:**
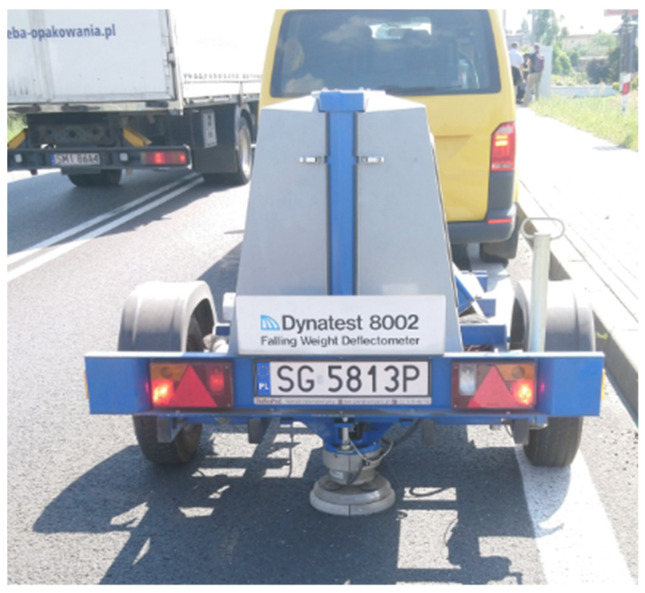
Pavement testing.

**Figure 3:**
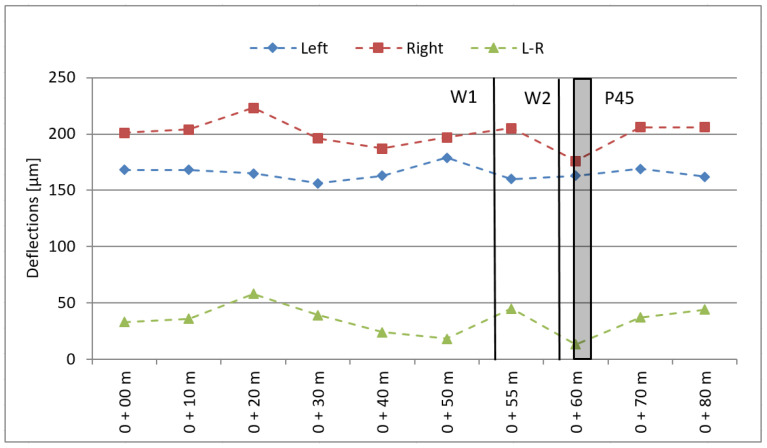
Pavement testing—dynamic deflections (W1, W2—strain gauge load cell sensors; P45—piezoelectric sensors).

In the next step, the dynamic deflection of the pavement was measured using Dynatest 8002 type Falling Weight Deflectometer (FWD) (Dynatest, Ballerup, Denmark) (Figure 2). The tests were carried out in August, with a mineral and asphalt layer temperature of +23 °C. The results obtained are demonstrated in Figure 3.

The calculated mean deflection values of 165 μm and 200 μm for the left and right wheel tracks, respectively, are values that classify the pavement as site I Excellent according to COST 323 for so-called “Flexible pavements”.

**Figure 4:**
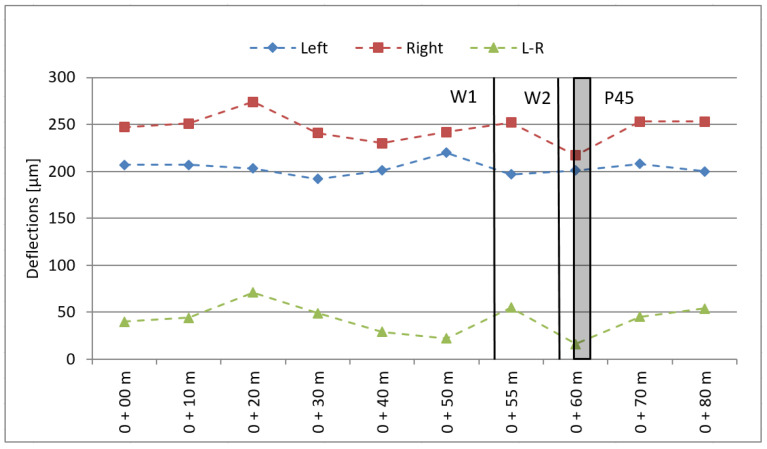
Pavement testing—static deflections (W1, W2—strain gauge load cell sensors; P45—piezoelectric sensors).

The pavement response to quasi-static loading was evaluated based on the results of the FWD deflectometer tests using a correlation coefficient between the FWD test and the Benkelman beam test (quasi-static loading) as reported by [22]. The test results are shown in Figure 4.

Under quasi-static loading conditions, the deflection of the pavement is less than 300 μm, whereas the difference in deflection for the left and right wheel tracks is less than 70 μm, which means that the pavement meets the requirements for flexible pavements and site I Excellent according to COST 323.

### 2.2. Sensors and Components

**Figure 5:**
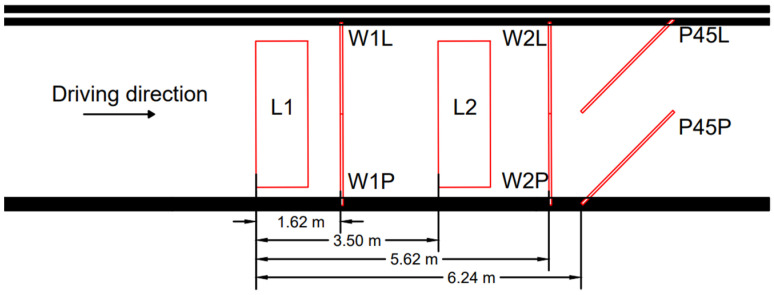
Sensor layout (L1, L2—loop sensors; W1L, W1P, W2L, and W2P—strain gauge load cell sensors; P45L, P45P—piezoelectric sensors).

The WIM station where the research was conducted consists of the following equipment (Figure 5):

- a set of strain gauges to measure the contact load on the right and left wheels of a given vehicle axle;
- a set of piezoelectric sensors to detect the tyre width, the tyre-sensor contact point and thus the lane position of the vehicle; and
- a set of inductive loops to trigger the reading of data from strain gauges and the determination of vehicle lengths.

**Figure 6:**
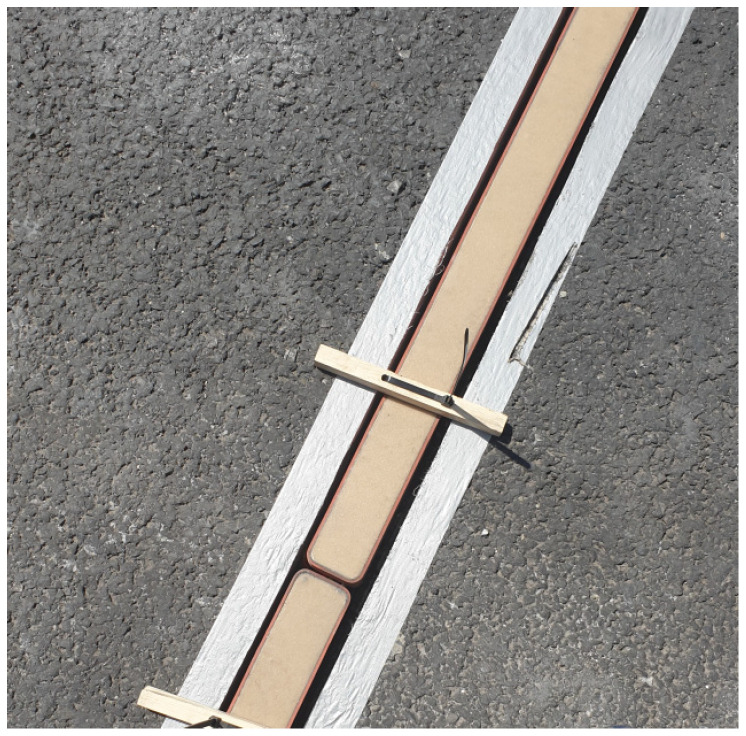
Strain gauge load cell sensors.

Linear strain gauge sensors constitute the main component of the weighing station. The installation used strain gauge load cell sensors (Intercomp, Medina, MN, USA) (Figure 6). They are constructed using resistance strain gauges that change their resistance due to strain. They have the following technical parameters [23]:

- length 1.75 m;
- sensor operating temperature from −40 to 80 °C;
- linearity < ±0.1% FSO; and
- temperature coefficient of sensitivity over the entire operating temperature range of ±0.0036%/°C.

**Figure 7:**
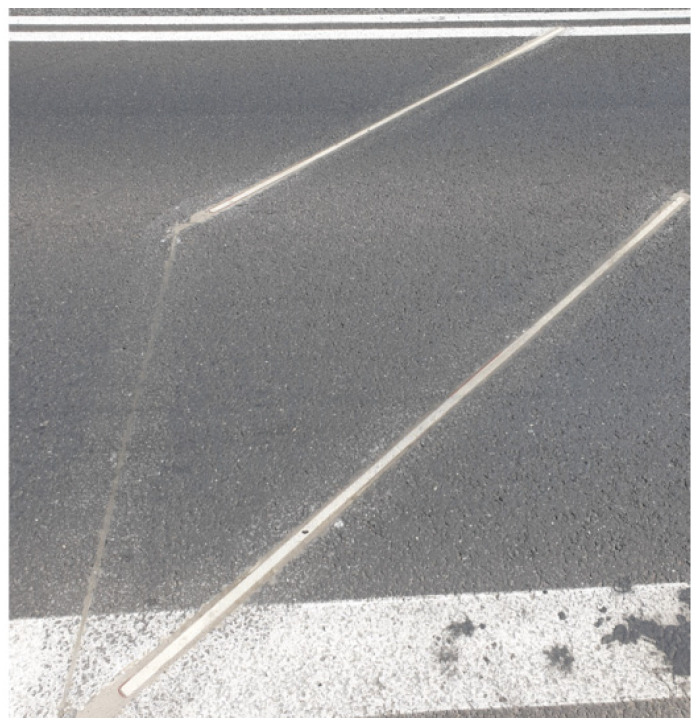
Piezoelectric Sensor.

The piezoelectric sensors used are ROADTRAX BL TRAFFIC SENSORs (TE Connectivity, Berwyn, PA, USA) (Figure 7). They have the following technical parameters [24]:

- length 2.5 m;
- sensor operating temperature from −40 to 70 °C;
- temperature sensitivity 0.2%/°C; and
- output uniformity ±20% for Class II.

**Figure 8:**
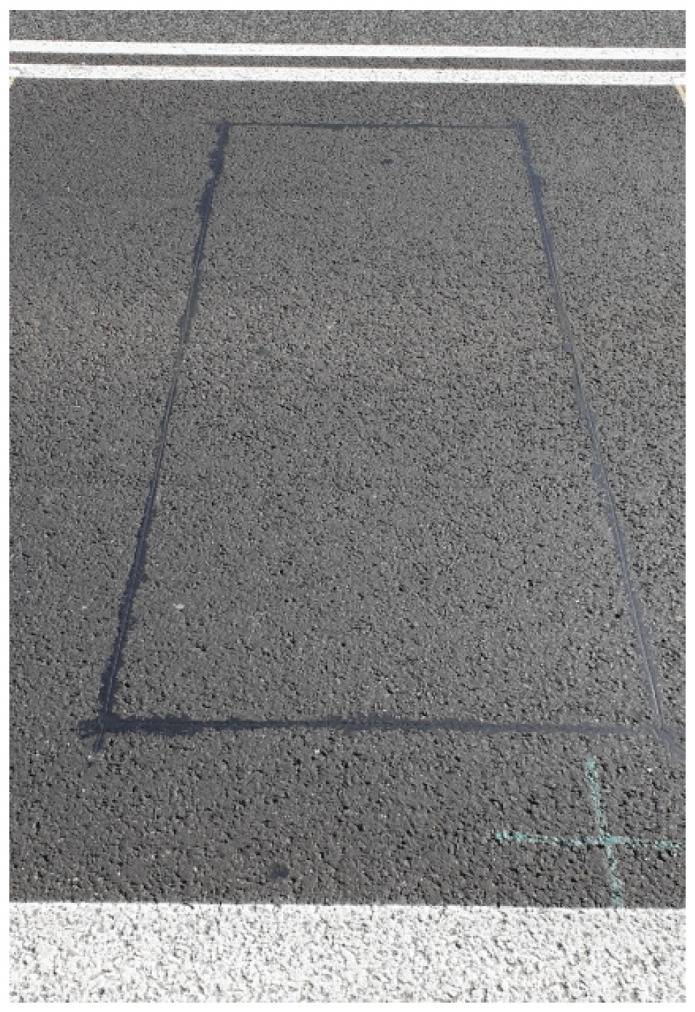
Loop sensors.

The induction loops were made according to the TLS 2012 specification [25] (Figure 8). They have the following technical parameters:

- size 1.0 × 2.8 m;
- 4 coils; and
- 1.5 mm^2^ copper wire.

**Figure 9:**
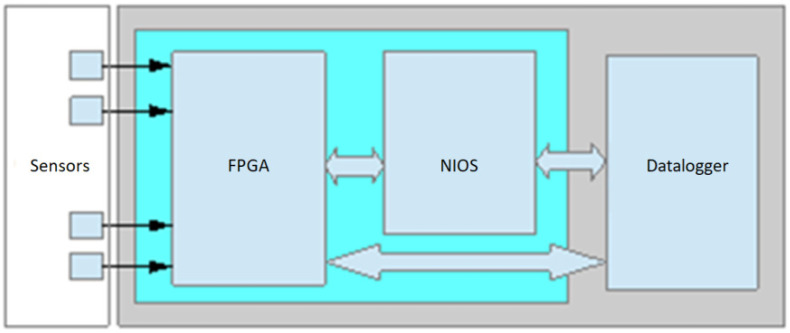
Block diagram of the WIM recorder.

The key component of the WIM system is a data logging device using analogue-to-digital tracks, FPGA (field-programmable gate array), NIOS (configurable embedded processor), and a datalogger built using an ARM controller (Figure 9). Using the FPGA-based architecture allows high performance in the processing, integration, and synchronization of signals from strain gauge load sensors, inductive loops, and additional piezoelectric sensors within a single device. The sampling frequency of WIM system is 31,250 Hz for strain gauge and piezoelectric sensors and 3125 Hz for inductive loops.

Each time, before starting a series of test runs, static loads were measured by means of a dedicated measuring station equipped with portable IRD SAW III scales of OIML R76 class (static accuracy ± 25 kg for weight up to 2.5 Mg; ± 50 kg for weight from 2.5 to 10 Mg).

### 2.3. System Calibration

**Figure 10:**
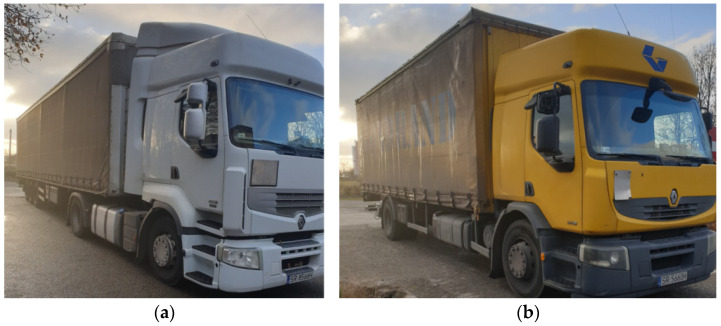
Vehicles used in the calibration exercise: (**a**) five-axle vehicle (category 5 acc. to COST 323), (**b**) two-axle vehicle (category 2 acc. to COST 323).

The system calibration was performed using a two-axle vehicle with a total weight of 18 Mg and a five-axle vehicle with a weight of 38 Mg (Figure 10). In the first stage, a number of test runs were conducted to select calibration coefficients separately for each strain gauge sensor. A series of 10 verification runs were then carried out to confirm the expected accuracy class of the measurement system. On each occasion, the vehicles were moving at the speed of approximately 50 km/h in the axis of the lane.

**Figure 11:**
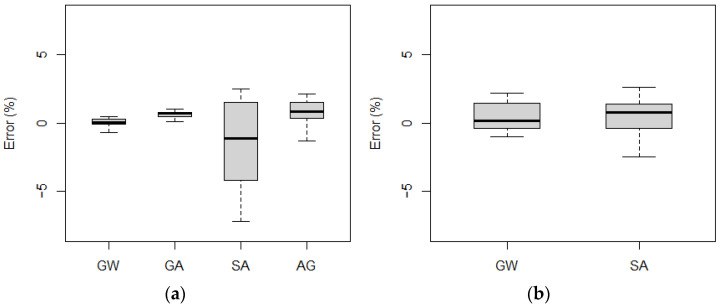
Obtained measurement errors for gross weight (GW), group of axles (GA), single axle (SA), and axle of a group (AG): (**a**) five-axle vehicle, (**b**) two-axle (the top and bottom of each box represent the 75th and 25th percentile values, whiskers show the minimum and maximum, the horizontal line represents the median).

Figure 11 shows the measurement error statistics obtained for each vehicle.

As can be observed, for the five-axle vehicle, the maximum gross weight measurement error did not exceed 1%, while for the two-axle vehicle it was 2%. The largest errors were recorded for the single axle measurement of the five-axle vehicle, with an error rate of 7% during a single pass. These quantities indicate that the system at the location in question meets the requirements of class A(5) according to COST 323.

## 3. Results and Discussion

### 3.1. Influence of the Trajectory on the Measurement Results

**Figure 12:**
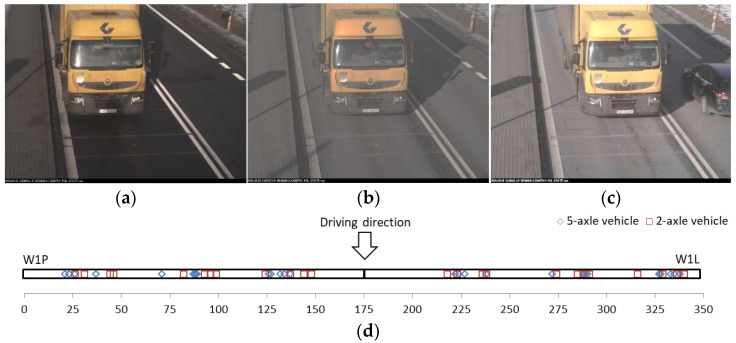
Control runs (**a**) right side, (**b**) central, (**c**) left side, and (**d**) position of tyre-sensor contact.

In the first series of tests, 14 runs each were made with a five-axle vehicle and a two-axle vehicle (Figure 12). The study involved the following three types of runs:

- right-side runs—at the edge of the site (Figure 12a);
- central runs—in the lane centreline (Figure 12b); and
- left-side runs—at the road axis (Figure 12c).

Each run was made at a speed of 50 km/h. The position of the vehicle was controlled using piezoelectric sensors (P45L/P45P) mounted at 45 degrees (examples for the first axle are shown in Figure 12d and in video analysis).

**Figure 13:**
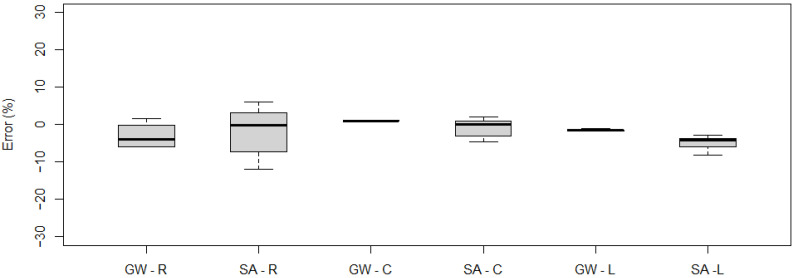
Measurement errors obtained for gross weight (GW), single axle (SA) for a five-axle vehicle: R—right-side run, C—central run, L—left-side run (the top and bottom of each box represent the 75th and 25th percentile values, whiskers show the minimum and maximum, the horizontal line represents median).

**Figure 14:**
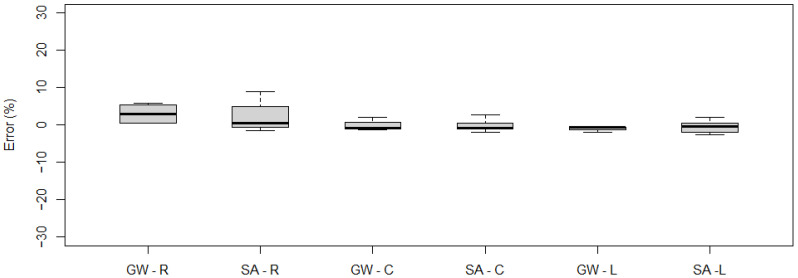
Measurement errors obtained for gross weight (GW), single axle (SA) for a two-axle vehicle: R—right-side run, C—central run, L—left-side run (the top and bottom of each box represent the 75th and 25th percentile values, whiskers show the minimum and maximum, the horizontal line represents median).

Figure 13 and Figure 14 demonstrate the measurement errors obtained for the gross weight and axle loads for the right and left sides and in the lane axis for the five-axle and two-axle vehicle, respectively.

The obtained results show that, in particular, the right side runs resulted in a measurement error that slightly exceeded the permissible values for A(5) class for both gross weight in the case of five-axle and two-axle vehicle and single axle load for five-axle vehicle. For the left-side runs, this error was comparable to the central runs.

**Figure 15:**
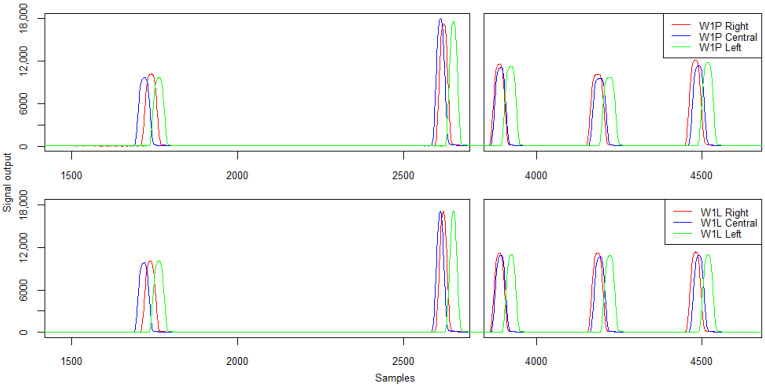
Example of signals from the first line of strain gauges for right- and left-side run and central—five-axle vehicle.

The following section presents example patterns of signals for a five-axle vehicle recorded during the tests. The signal patterns from the strain gauge sensors (W1P and W1L) are demonstrated in Figure 15. The characteristics of the recorded signals with respect to the amplitude and calculated load of individual vehicle wheels are presented in Table 4 and Table 5. The coefficient R² between the signal amplitude and the wheel load was 0.79 for sensor W1P and 0.85 for W1L.

The analysis of the presented characteristics and signal patterns indicates a change in the amplitude and load recorded for each wheel by strain gauges W1P and W1L in the case of passing on the right side of the lane. In the case of axle 3, 4, and 5, the error exceeded 10%. Importantly, laboratory tests of sensor linearity performed in previous work did not indicate significant changes in signal values depending on the load position [26].

**Figure 16:**
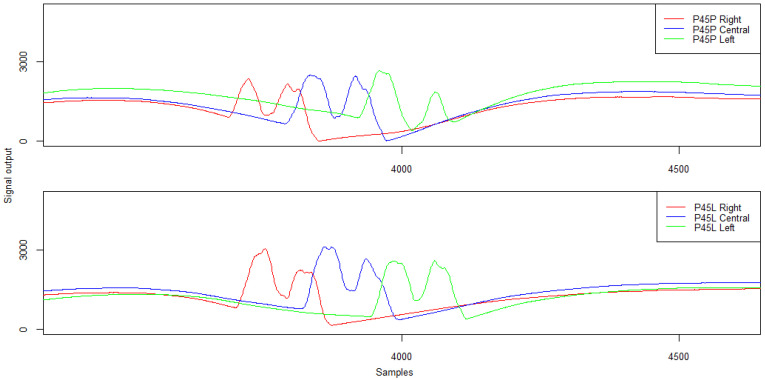
Example of signals from the piezoelectric sensors for right- and left-side run and in lane alignment—the second axis of the five-axle vehicle.

The driving trajectory, in the case of piezoelectric sensors P45P and P45L, has also a slight impact on the signal corresponding to the recording of the load from the twin wheel. The signal patterns for the twin wheel at different passing modes are shown in Figure 16 and their characteristics in Table 6.

The analysis of the waveform and characteristics of the signal recorded by the P45P sensor for the right-side lane run indicates a comparable amplitude value and a correct response of the sensor to the load from the twin wheel. Only for the left-side run is the Peak–Peak distance slightly higher. However, in all the cases, the identification of the twin wheel is possible.

**Figure 17:**
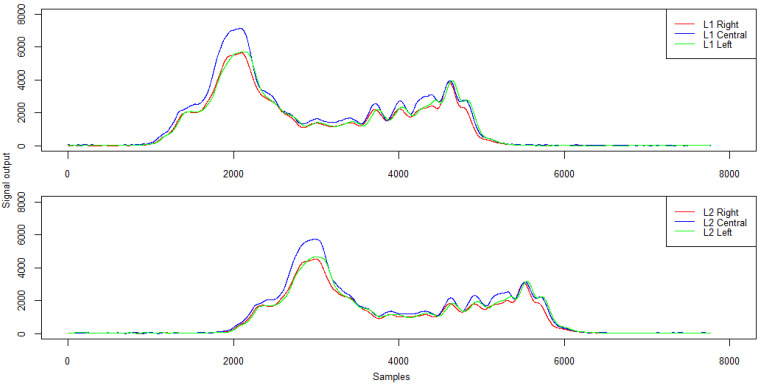
Example signals from induction loops for right- and left-hand passing and in lane alignment—five-axle vehicle.

Changing the trajectory also causes some differences in the signals recorded by the induction loops. Example signals from induction loops for right- and left-side passing and in lane alignment for a five-axle vehicle are presented in Figure 17. The characteristics of the signal from the induction loops are given in Table 7.

For the analysed trajectories, both loop L1 and loop L2 generated a qualitatively similar signal to ensure that the measurement path of the WIM station was properly activated and the vehicle length was correctly calculated. However, the passing trajectory affected the peak value and the mean signal level which reach the highest values when passing in the lane centreline (normative) and the lowest when passing on the right side of the lane.

The analysis of the measurement errors for the different trajectories indicates that passing on the right side of the lane could result in an increase in the measurement error exceeding the acceptable level. In the authors’ opinion, especially in WIM systems with a direct enforcement function, it is worth considering monitoring the trajectories, e.g., by determining the tyre-sensor contact point using piezoelectric sensors mounted at 45 degrees.

### 3.2. Effects of Acceleration/Deceleration on Measurement Results

As part of the research on the influence of the runs’ dynamics on the sensor signals at the WIM station, 12 runs were made with a five-axle vehicle and 12 runs with a two-axle vehicle. Three types of runs were included in the study:

- central runs at a constant speed of 50 km/h;
- central runs with a change in speed (accelerating)—vehicle driving 30 km/h and then accelerating approximately 30 m before the set of measurement sensors (accelerating range between 0.49 and 0.61 m/s²); and
- central runs with a change in speed (braking)—vehicle driving 50 km/h and then braking through a set of measurement sensors (accelerating range between −1.86 and −0.68 m/s²).

**Figure 18:**
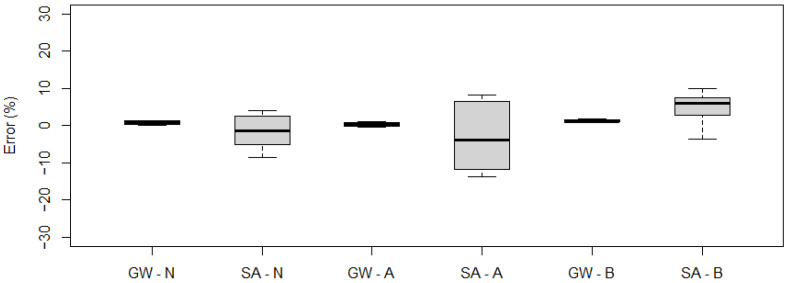
Measurement errors obtained for gross weight (GW), single axle (SA) for a five-axle vehicle: N—runs at constant speed, A—accelerating, and B—braking (the top and bottom of each box represent the 75th and 25th percentile values, whiskers show the minimum and maximum, the horizontal line represents the median).

**Figure 19:**
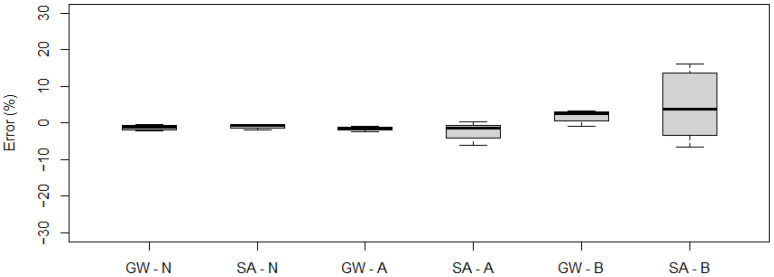
Measurement errors obtained for gross weight (GW), single axle (SA) for a two-axle vehicle: N—runs at constant speed, A—accelerating, and B—braking (the top and bottom of each box represent the 75th and 25th percentile values, whiskers show the minimum and maximum, the horizontal line represents the median).

Figure 18 and Figure 19 show the measurement errors obtained for the gross weight and axle loads for constant speed, acceleration, and braking runs.

The results obtained indicated a fairly significant scatter of values for the single axle load measurement of a five-axle vehicle (Figure 18). For the two-axle vehicle, significant errors (>10%) were recorded for the passage with braking (Figure 19).

**Figure 20:**
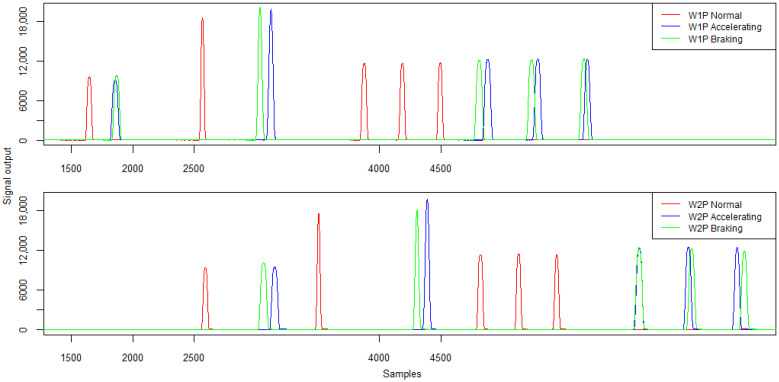
Example signals from the strain gauges sensors W1P and W2P of strain gauges for constant speed, acceleration and braking—five-axle vehicle.

Example waveforms of signals from strain gauges concerning the passage of a five-axle vehicle are shown in Figure 20. Table 8 and Table 9 summarise the signal characteristics in relation to the signal amplitude and the calculated load of the individual vehicle wheels. Table 9 also contains information on the static wheel loads. For this type of run, due to the dynamics, it was decided to present the signals from two sensor lines measuring the wheel load of the right side of the vehicle (signal W1P and W2P).

The analysis of the values of signal amplitudes obtained during the run with acceleration or braking in the WIM station area indicates an average difference of more than 6% in the values of amplitudes in relation to the run with constant speed. In the vast majority of cases, a dynamic run results in a higher amplitude signal being recorded for individual vehicle wheels. When analysing the load values for the wheel of the first axle in the case of a run with deceleration, it can be easily observed that it is overloaded compared to a constant-speed run and that it is under-loaded in the case of an accelerated run. These variations are within ±15%.

**Figure 21:**
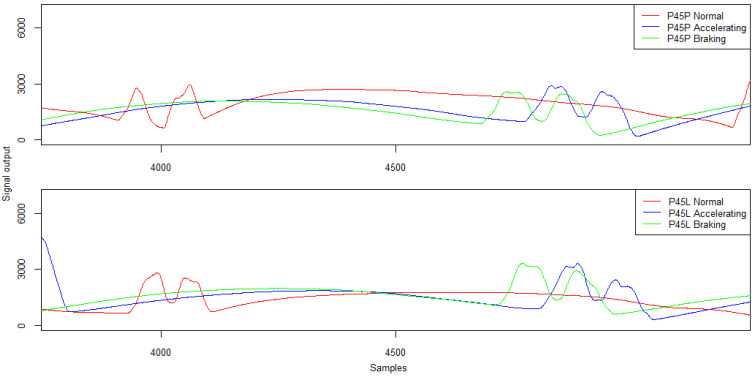
Example signals for the twin wheel from the piezoelectric sensors at constant speed, acceleration, and braking—five-axle vehicle.

The signal patterns for the twin wheel during the variable speed run are shown in Figure 21 and their characteristics in Table 10.

The change of speed when passing through piezoelectric sensors causes, in most cases, an increase of several per cent in the signal amplitude value. The average difference in amplitude values was almost 12%. It is worth noting, however, that variable speed runs lead to the effect of a specific stretching of the signal in the vicinity of the maximum value, which causes problems in the unambiguous determination of the Peak–Peak distance and, consequently, may cause problems in the identification of the twin wheel.

**Figure 22:**
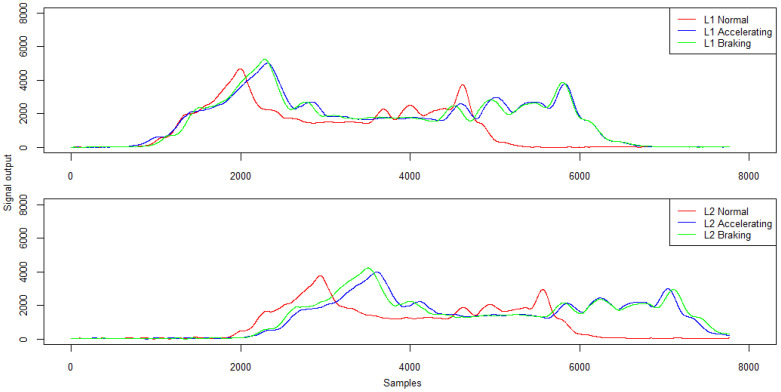
Example signals from the induction loops at constant speed, acceleration and braking—five-axle vehicle.

Example signals from induction loops for a variable speed run for a five-axle vehicle are presented in Figure 22. The characteristics of the signal from the induction loops are given in Table 11.

The signal recorded by the induction loop during variable speed runs is qualitatively consistent with the signal for a constant speed run. However, during such runs the maximum value of the signal is on average more than 9% higher. Also, the mean value of the recorded signal for runs with a variable speed is several per cent higher on average. Nevertheless, this does not have a major impact on the correctness of the determined vehicle length. For this parameter, the maximum error reaches 2%.

The analysis of measurement errors for variable speed runs shows that accelerating or braking while passing through the measurement system can result in an increase in axle load measurement error beyond the acceptable level. However, the system consisting of duplicate sensors allows the gross weight to be determined correctly.

### 3.3. Influence of Trajectory Changes on the Measurement Results

**Figure 23:**
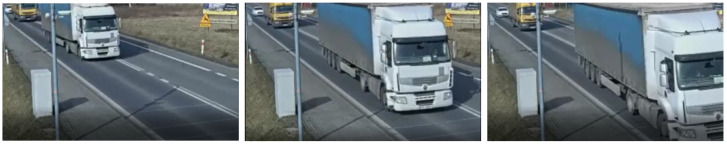
Example of a run with a change of trajectory.

The last element of the study was to perform runs with a change of trajectory. In this case, the vehicles initially travelled on the left side of the lane and then, when passing the sensor set, made a change of trajectory towards its right side (Figure 23).

Taking into account the results obtained for all trajectory-changing runs performed, the average error in determining the gross weight, the individual axle load and the group of axles was estimated. The results are summarised in Table 12.

The analysis of the data presented in Table 12 allows us to conclude that a change of trajectory when a WIM station is equipped with a set of duplicate sensors does not cause the exceedance of the permissible error for a class A(5) station. However, it is noteworthy that in this case the error in axle load may be several times greater than the error in determining the gross weight.

**Figure 24:**
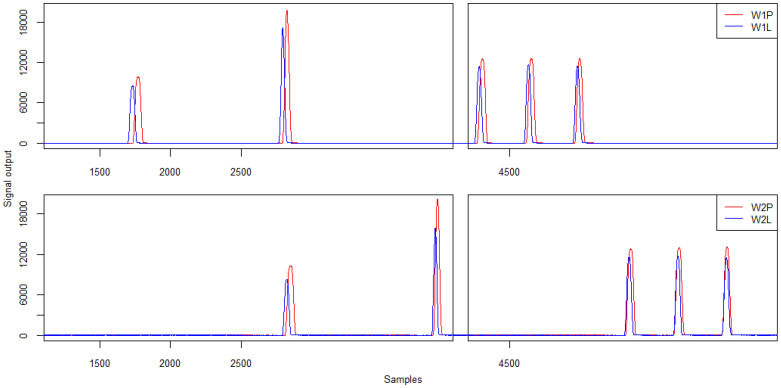
Example signals from strain gauges for changing the trajectory of a five-axle vehicle.

Examples of signal patterns from strain gauge sensors are shown in Figure 24. In this case, the signals for the left and right sides of the first (W1P, W1L) and second lines (W2P, W2L) are collated.

Table 13 and Table 14 summarise the signal characteristics in relation to the signal amplitude and the calculated load of the individual vehicle wheels.

The analysis of the signal amplitude values from sensors recording the same vehicle wheel passage (i.e., W1P and W2P, and W1L and W2L, respectively) reveals that higher amplitude values are obtained for sensors located on the right side. Also, when comparing the loads for individual wheels on the left and right side of the vehicle, the values for the wheels on the right side of the vehicle were several per cent higher. While analysing the test’s video recordings, it was found that drivers started the manoeuvre of changing their lane clearly before the WIM station. In the measuring area, the manoeuvre was in its final phase, i.e., the vehicle was making a left turn. According to the principles of dynamics, it is the right wheels that are under greater strain at this time. Furthermore, by analysing the amplitude shifts of the signals for the left and right wheels of the vehicle, respectively, it is possible to infer a change in the path of the vehicle passing through the measuring station.

**Figure 25:**
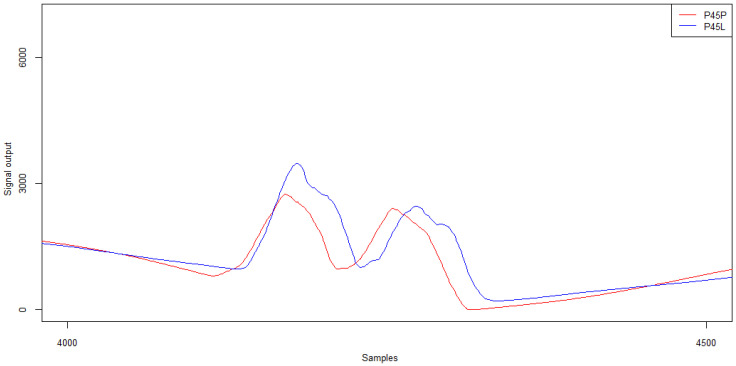
Example of piezoelectric sensor signals for the twin wheel for trajectory changes for a five-axle vehicle.

The signal waveforms for the twin wheel for the trajectory change are shown in Figure 25 and their characteristics in Table 15.

Changing the trajectory when passing through the piezoelectric sensors resulted in a change in the value of the signal amplitude which was greater for sensor P45L, thus located on the left side. The change in trajectory also affected the Peak–Peak distance, but to an extent that did not cause major problems in identifying the twin wheel.

**Figure 26:**
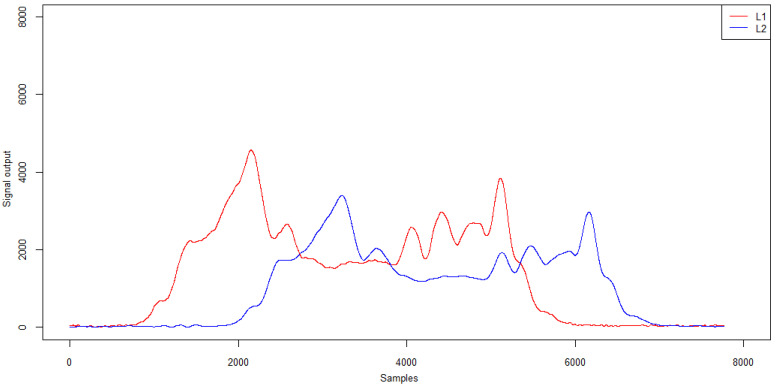
Example of induction loop signals for trajectory changes for a five-axle vehicle.

An example of induction loop signals for a variable path run is presented in Figure 26. The characteristics of the signal from the induction loops are given in Table 16.

Trajectory changing has an effect on the variation of the signal recorded by the induction loops. The signals recorded by both loops are qualitatively consistent, but a significantly weaker signal was recorded by the second loop L2. However, this does not increase the error in determining the vehicle length. The average relative error is still in the range of 3–4%.

## 4. Conclusions

The practical use of WIM systems for direct enforcement will require them to be highly reliable in terms of the data they record. It is worth noting that currently even the most modern WIM stations do not achieve 100% efficiency [27]. In this paper, attention has been paid to the influence of both the trajectory and the dynamics of driving through a WIM station on the signals generated by the sensors. The patterns that caused errors exceeding the acceptable accuracy for WIM class A(5) stations or signal deformations that prevented proper interpretation were recorded.

Based on the research presented in this paper, the following conclusions have been drawn:

- passing on the right side of the lane (i.e., close to the road end) could result in an increase in the measurement error exceeding the acceptable level;
- accelerating or braking while passing through the measurement system can result in an increase in axle load measurement error beyond the acceptable level;
- changing the trajectory when a WIM station is equipped with a set of duplicate sensors does not cause the exceedance of the permissible error for a class A(5) station; and
- for the study conducted, there were no above-normal errors associated with the determination of vehicle length by means of induction loops.

In addition, attention should be paid to non-central runs, i.e., those where the tyre could only partially overlap the load cell. Such cases may occur at most locations, as it is very rare that solutions delineating the correct trajectory, such as the use of traffic separators, are implemented in the WIM station area. It is obvious that such runs must be detected by the direct enforcement system as abnormal runs and consequently rejected.

The manner and dynamics of driving through a WIM station are not the only factors that may disqualify a given measurement as a basis for direct enforcement. The paper also presents the measurements of pavement quality which are necessary to be able to assign a WIM station to the appropriate accuracy class. Such measurements shall be repeated periodically to confirm that the pavement meets the requirements of COST 323 despite natural deterioration. The failure of the road pavement to meet the quality requirements is tantamount to excluding the WIM station from operation.

Other factors that can discredit a measurement for direct enforcement are meteorological factors. This includes the temperature of the roadway and its base, the condition of the roadway (dry, ice, or snow) as well as the direction and the speed of wind. Assessing the impact of these factors on the suitability of the measurement for direct enforcement undoubtedly requires further in-depth research.

## Figures and Tables

**Table 1:** Location parameters.

Parameter	Value
ADDT [veh./day]	17,176 ^1^
HVs [%]	9 ^1^
Lane width [m]	3.5

^1^ General Traffic Measurement 2015.

**Table 2:** WIM accuracy classes. Data from [21].

Criteria	Accuracy Classes: Confidence Interval Width δ (%)					
	A(5)	B+(7)	B(10)	C(15)	D+(20)	D(25)
Gross weight > 3.5 t	5	7	10	15	20	25
Axle load of group of axles > 1 t	7	10	13	18	23	28
Axle load of single of axles > 1 t	8	11	15	20	25	30
Axle load of axle of a group > 1 t	10	14	20	25	30	35
Minimum WIM site class	I	I	II	III	III	III

**Table 3:** Pavement parameters.

Parameter	Value
Longitudinal gradient of the road (grade line)	0.7%
Transverse slope (slope)	2.62%
Longitudinal evenness of pavement	0.66 mm/m
Cross evenness of the pavement (track depth)	1 mm (left) 4 mm (right)

**Table 4:** Strain gauge sensor signal characteristics—signal amplitude in Analog-Digital Unit (ADU).

**Run/Sensor W1P**	**Wheel 1**	**Wheel 2**	**Wheel 3**	**Wheel 4**	**Wheel 5**
Right	10,163	17,198	11,564	10,095	12,132
Central	9618	18,040	11,071	9525	11,317
Left	9610	17,559	11,222	9694	11,815
Run/Sensor W1L	Wheel 1	Wheel 2	Wheel 3	Wheel 4	Wheel 5
Right	10,109	17,072	11,208	11,222	11,356
Central	9834	17,088	10,899	10,691	10,906
Left	10,077	17,094	11,007	10,844	10,993

**Table 5:** Strain gauge sensor signal characteristics and reference static measurements—wheel load (kg).

**Run/Sensor W1P**	**Wheel 1**	**Wheel 2**	**Wheel 3**	**Wheel 4**	**Wheel 5**
Static	3450	4650	3550	3700	3950
Right	3731	4766	4043	4114	4399
Central	3343	4886	3677	3779	4030
Left	3244	4653	3918	3707	4080
Run/Sensor W1L	Wheel 1	Wheel 2	Wheel 3	Wheel 4	Wheel 5
Static	3450	4550	3350	3600	3600
Right	3479	4487	3801	4017	3955
Central	3349	4370	3614	3635	3634
Left	3467	4365	3461	3649	3598

**Table 6:** Piezoelectric sensor signal characteristics—amplitude (ADU) and Peak–Peak separation distance (samples) for a twin wheel.

**Run/Sensor P45P**	**Peak 1**	**Peak 2**	**Peak–Peak Distance**
Right	2344	2164	70
Central	2502	2453	81
Left	2664	1849	101
Run/Sensor P45L	Peak 1	Peak 2	Peak–Peak Distance
Right	3038	2235	63
Central	3115	2667	76
Left	2578	2590	74

**Table 7:** Signal characteristics from inductive loops—amplitude and mean signal level (ADU).

**Run\Loop L1**	**Max**	**Mean**
Right	4775	1686
Central	7128	2217
Left	5696	2050
Run\Loop L2	Max	Mean
Right	3870	1328
Central	5730	1942
Left	4652	1639

**Table 8:** Strain gauge sensor signal characteristics—signal amplitude (ADU).

**Run/Sensor W1P**	**Wheel 1**	**Wheel 2**	**Wheel 3**	**Wheel 4**	**Wheel 5**
Normal	9520	18,538	11,614	11,653	11,700
Accelerating	9105	19,817	12,212	12,292	12,247
Braking	9759	20,021	12,105	12,173	12,306
Run/Sensor W2P	Wheel 1	Wheel 2	Wheel 3	Wheel 4	Wheel 5
Normal	9363	17,521	11,355	11,420	11,286
Accelerating	9506	19,770	12,341	12,473	12,413
Braking	10,125	18,065	12,229	12,267	11,928

**Table 9:** Strain gauge sensor signal characteristics and reference static measurements—wheel load (kg).

**Run/Sensor W1P**	**Wheel 1**	**Wheel 2**	**Wheel 3**	**Wheel 4**	**Wheel 5**
Static	3500	4600	4050	4200	3900
Normal	3474	4787	4391	4191	4046
Accelerating	3086	5038	4476	4271	3987
Braking	3607	5111	4240	4286	4178
Run/Sensor W2P	Wheel 1	Wheel 2	Wheel 3	Wheel 4	Wheel 5
Static	3500	4600	4050	4200	3900
Normal	3248	4262	4254	3956	3642
Accelerating	3198	4947	4346	4169	3865
Braking	3739	3917	4295	3965	3656

**Table 10:** Piezoelectric sensor signal characteristics—amplitude (ADU) and Peak–Peak separation distance (samples) for a twin wheel.

**Run/Sensor P45P**	**Peak 1**	**Peak 2**	**Peak–Peak Distance**
Normal	2767	2959	112
Accelerating	2887	2585	107
Braking	2572	2466	117
Run/Sensor P45L	Peak 1	Peak 2	Peak–Peak Distance
Normal	2828	2542	58
Accelerating	3324	2444	81
Braking	3341	2925	113

**Table 11:** Signal characteristics from inductive loops—amplitude and mean signal value (ADU).

**Run\Loop L1**	**Max**	**Mean**
Normal	4686	1833
Accelerating	5028	1996
Braking	5242	2038
Run\Loop L2	Max	Mean
Normal	3755	1427
Accelerating	3985	1680
Braking	4231	1762

**Table 12:** Results for runs with a change of trajectory.

**Mean [%]**	**GW**	**Axle 1**	**Axle 2**	**Axle 3**	**Axle 4**	**Axle 5**	**GA**
five-axle	0.8%	4.9%	3.9%	4.5%	2.2%	1.7%	1.4%
two-axle	1.1%	2.6%	1.1%	-	-	-	-

**Table 13:** Strain gauge sensor signal characteristics—signal amplitude (ADU).

**Sensor/Wheel**	**Wheel 1**	**Wheel 2**	**Wheel 3**	**Wheel 4**	**Wheel 5**
W1P	9851	19,703	12,517	12,559	12,604
W1L	8494	17,201	11,406	11,610	11,467
Sensor/Wheel	Wheel 1	Wheel 2	Wheel 3	Wheel 4	Wheel 5
W2P	10,340	20,203	12,833	13,001	13,104
W2L	8306	15,943	11,682	11,801	11,512

**Table 14:** Strain gauge sensor signal characteristics—wheel load (kg).

**Sensor /Wheel**	**Wheel 1**	**Wheel 2**	**Wheel 3**	**Wheel 4**	**Wheel 5**
Static P	3500	4600	4050	4200	3900
Static L	3350	3850	3650	3850	3850
W1P	3548	5338	4590	4584	4337
W1L	2666	4012	3461	3445	3463
Sensor/Wheel	Wheel 1	Wheel 2	Wheel 3	Wheel 4	Wheel 5
Static P	3500	4600	4050	4200	3900
Static L	3350	3850	3650	3850	3850
W2P	3841	5312	4721	4721	4555
W2L	2331	3278	3440	3278	3206

**Table 15:** Piezoelectric sensor signal characteristics—amplitude (ADU) and Peak–Peak separation distance (samples) for a twin wheel.

Sensor/Parameter	Peak 1	Peak 2	Peak–Peak Distance
P45P	2743	2411	84
P45L	3475	2468	94

**Table 16:** Induction loop signal characteristics—amplitude and mean signal value (ADU).

**Loop/Parameter**	**Max**	**Mean**
L1	4569	1944
L2	3400	1498

## Data Availability

The data presented in this study are available on request from the corresponding author. The data are not publicly available due to the size of the dataset.
